# Antibacterial efficacy and membrane mechanism of action of the *Serratia*-derived non-ionic lipopeptide, serrawettin W2-FL10

**DOI:** 10.1128/spectrum.02952-23

**Published:** 2024-06-06

**Authors:** Tanya Decker, Marina Rautenbach, Sehaam Khan, Wesaal Khan

**Affiliations:** 1 Water Health Research Centre, Faculty of Health Sciences, University of Johannesburg, Doornfontein, Johannesburg, Gauteng, South Africa; 2 BioPep Peptide Group, Department of Biochemistry, Faculty of Science, Stellenbosch University, Stellenbosch, South Africa; 3 Department of Microbiology, Faculty of Science, Stellenbosch University, Stellenbosch, South Africa; Universidade de Brasilia, Brasilia, Brazil

**Keywords:** serrawettin W2, lipopeptide, antibacterial, mode of action, cytoplasmic membrane

## Abstract

The study aimed to investigate the antibacterial activity, cytotoxicity, and mechanism of action of the non-ionic, cyclic lipopeptide, serrawettin W2-FL10 against *Staphylococcus aureus*. W2-FL10 exhibited potent activity against the Gram-positive bacteria *S. aureus*, *Enterococcus faecalis*, *Enterococcus faecium*, *Listeria monocytogenes,* and *Bacillus subtilis*, with minimum inhibitory concentration (MIC) values ranging from 6.3 to 31.3 μg/mL, while no activity was observed against Gram-negative bacteria. Broth microdilution assays showed that W2-FL10 interacted with key cell membrane components, such as lipid phosphatidyl glycerol and lipoteichoic acid of *S. aureus*. Upon membrane interaction, W2-FL10 dissipated membrane potential within 12 min and increased *S. aureus* membrane permeability within 28–40 min, albeit at slower rates and higher concentrations than the lytic peptide melittin. The observed membrane permeability, as detected with propidium iodide (PI), may be attributed to transmembrane pores/lesions, possibly dependent on dimer-driven lipopeptide oligomerization in the membrane. Scanning electron microscopy (SEM) imaging also visually confirmed the formation of lesions in the cell wall of one of the *S. aureus* strains, and cell damage within 1 h of exposure to W2-FL10, corroborating the rapid time-kill kinetics of the *S. aureus* strains. This bactericidal action against the *S. aureus* strains corresponded to membrane permeabilization by W2-FL10, indicating that self-promoted uptake into the cytosol may be part of the mode of action. Finally, this lipopeptide exhibited low to moderate cytotoxicity to the Chinese hamster ovarian (CHO) cell line in comparison to the control (emetine) with an optimal lipophilicity range (log D value of 2.5), signifying its potential as an antibiotic candidate.

**IMPORTANCE:**

Antimicrobial resistance is a major public health concern, urgently requiring antibacterial compounds exhibiting low adverse health effects. In this study, a novel antibacterial lipopeptide analog is described, serrawettin W2-FL10 (derived from *Serratia marcescens*), with potent activity displayed against *Staphylococcus aureus*. Mechanistic studies revealed that W2-FL10 targets the cell membrane of *S. aureus*, causing depolarization and permeabilization because of transmembrane lesions/pores, resulting in the leakage of intracellular components, possible cytosolic uptake of W2-FL10, and ultimately cell death. This study provides the first insight into the mode of action of a non-ionic lipopeptide. The low to moderate cytotoxicity of W2-FL10 also highlights its application as a promising therapeutic agent for the treatment of bacterial infections.

## INTRODUCTION

Antimicrobial resistance poses a significant global public health concern (1). Key to addressing this threat is the discovery of antibiotics that have novel modes of action, with prolonged therapeutic timelines (2). In the last six decades, only two new classes of antibiotics with unique modes of action have been introduced onto the market, one of which (i.e., daptomycin) belongs to the lipopeptide class of antibiotics (3).

Lipopeptides represent a class of low molecular weight metabolites that are synthesized non-ribosomally by various bacteria and fungi (4). Structurally, they consist of a hydrophobic lipid or fatty acid moiety covalently linked to the N-terminus of a linear or cyclic hydrophilic peptide, which can be ionic (anionic or cationic) or non-ionic (4, 5). *Serratia* species have been highlighted as promising sources of antibacterial lipopeptides, with a study by our research group expanding on the structures of the serrawettin W2 family (6). Members of the serrawettin W2 family are non-ionic, cyclic lipopeptides produced by *Serratia marcescens* and *Serratia surfactantfaciens* strains (6–8). Serrawettin W2 lipopeptides are comprised of five amino acid residues (D-Leu-L-Ser-L-Thr-D-Phe/Trp/Tyr/Leu/Ile-L-Ile/Leu/Val), connected to a β-hydroxy fatty acid moiety (chain length of C_8_, C_10_, C_12_, or C_12:1_; molecular weight range of 690 to 771 Da). Currently, 24 analogs of serrawettin W2 have been identified and the nomenclature of analogs (i.e., W2-FI10, W2-YV10, W2-W(L/I)10 or W2-FV12:1, amongst many others) was recently clarified (6).

**Fig 1:**
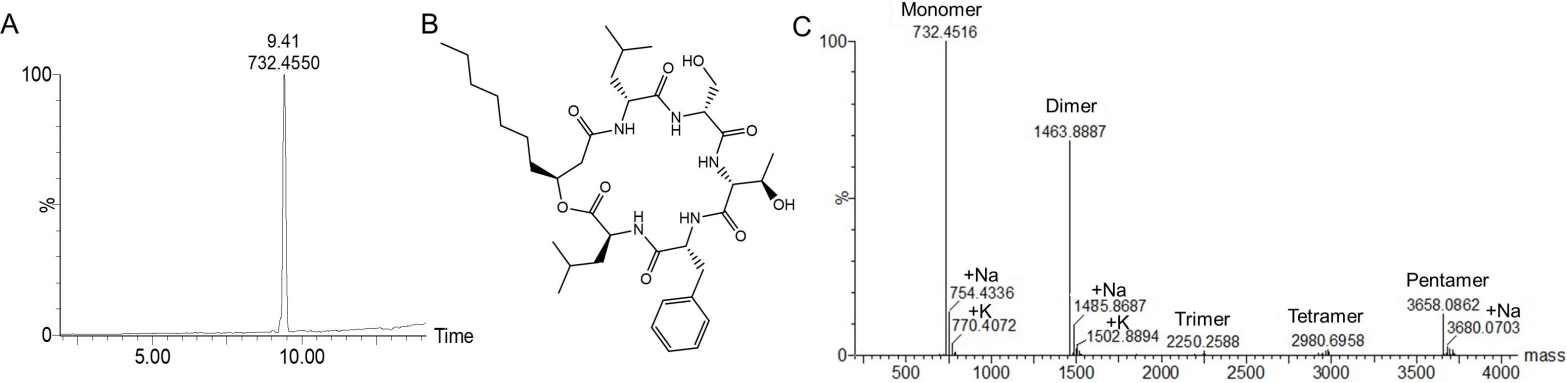
The ultra-performance liquid chromatography coupled to high-resolution tandem mass spectrometry (UPLC-HRMS) analysis of the reverse-phase high-performance liquid chromatography (RP-HPLC) purified W2-FL10 fraction (**A**), the corresponding primary structure of serrawettin W2-FL10 (**B**), and MaxEnt 3 mass spectrum of the purified W2-FL10 showing its tendency to oligomerize (**C**).

The antimicrobial activity of the serrawettin W2-FI10 analog (i.e., C_10_H_18_O_2_-Leu-Ser-Thr-Phe-Ile), has been extensively investigated. For example, Su et al. (8) demonstrated that W2-FI10, produced by *S. surfactantfaciens* sp. nov. YD25^T^, displayed activity against Gram-negative and Gram-positive bacteria, such as *Pseudomonas aeruginosa*, *Shigella dysenteriae,* and *Staphylococcus aureus*. In addition, Heise et al. (9) isolated an *S. marcescens* 2MH3-2 strain that produced W2-FI10 and showed that it displayed inhibitory activity against methicillin-resistant *S. aureus* (MRSA), methicillin-susceptible *S. aureus* (MSSA), *Listeria monocytogenes,* and *Bacillus subtilis* at 4 µg/mL. Recently, Clements-Decker et al. (6) found that the novel analog, W2-FL10 (i.e., C_10_H_18_O_2_-Leu-Ser-Thr-Phe-Leu; Fig. 1), which differs from W2-FI10 based on the amino acid change of Ile to Leu at the fifth residue, exhibited potent activity against *Enterococcus faecium* [minimum inhibitory concentration (MIC) of 15.6 µg/mL].

Detailed elucidation of the mode of action of lipopeptides remains under investigation; however, key mechanistic properties have been identified. For example, various lipopeptides bind to and are inserted into the bacterial membrane based on electrostatic (charge of the peptide moiety) and hydrophobic (due to the lipid tail) interactions (5). Following insertion, many lipopeptides oligomerize within the bacterial membrane, forming oligomeric transmembrane pores which lead to membrane depolarization (perforation of ions) and/or permeability of the membrane (leakage of intracellular components) (5, 10). However, the broad-spectrum activity and mode of action of W2-FL10 remains unknown.

This study aimed to explore the antibacterial activity of WL-FL10, and subsequently investigate the mode of action of this lipopeptide against *S. aureus*. To assess the antibacterial properties of W2-FL10, broth microdilutions were conducted with variations including the presence or absence of cations, lipoteichoic acid (LTA) and 1,2-dioleoyl-sn-glycero-3-phospho-(1'-rac-glycerol) (DOPG). Time-kill assays were performed to elucidate the bactericidal or bacteriostatic effects of W2-FL10 over time, providing insight into its efficacy and kinetics of action. Furthermore, the membrane-targeting potential of the lipopeptide was explored through membrane permeabilization and depolarization assays. Morphological impacts on the cell wall were examined using scanning electron microscopy (SEM). Finally, the cytotoxicity, lipophilicity, and solubility of W2-FL10 were evaluated.

## RESULTS

### W2-FL10 chemical properties

The lipopeptide, W2-FL10, was purified using reverse-phase high-performance liquid chromatography (RP-HPLC), and the purity of the collected W2-FL10 fraction (experimental *m/z* of 732.4550 [M+H]^+^, expected 732.4548) was determined as 95% pure (Fig. 1A) using ultra-performance liquid chromatography coupled to high-resolution tandem mass spectrometry (UPLC-HRMS^E^). Clements-Decker et al. (6) confirmed that this neutral lipopeptide’s amino acid sequence was D-Leu-L-Ser-L-Thr-D-Phe-D-Leu, with an ether bond between the C_10_H_18_O_2_ fatty acyl’s β-hydroxyl group and L-Leu, and an amide bond between the fatty acyl carboxyl and D-Leu to form a cyclic lipopeptide (Fig. 1B). To evaluate and characterize the medically relevant physiochemical properties of this drug candidate, a number of assays were performed, including log D and solubility determination. The log D parameter of W2-FL10 was within the optimal lipophilicity range for oral absorption and cell membrane permeation at 2.5. However, W2-FL10 was found to exhibit a poor solubility of lower than 5 µM at pH 7.4 in water. This low solubility may be due to the high tendency of this peptide to form oligomers (i.e., dimers, trimers, tetramers, and pentamers), which were detected with electrospray Ionization (ESI)-HRMS (Fig. 1C). As hydrophobic interactions are negligible in the *in vacuo* high energy environment of an MS, only the strongest electrostatic interactions (i.e., hydrogen bonds) are involved in the detected oligomers (11–14). This is indicative of specific interactions between the peptide backbone, which may be important in the active structure(s).

### Antibacterial activity and mammalian cell cytotoxicity of W2-FL10

To assess the antibacterial properties of W2-FL10, a standard broth microdilution assay was performed to determine the half-maximal inhibitory concentration (IC_50_; 50% growth inhibition) and minimum inhibitory concentration (MIC; ≥90% growth inhibition) (15) of W2-FL10 against a panel of Gram-negative and Gram-positive bacterial strains. This assay was conducted in comparison to an antimicrobial standard, melittin (85% purity), a lytic antimicrobial peptide from bee venom that was included in this study as a positive control (16). The potent activity was observed for W2-FL10 against several Gram-positive bacteria (Table 1), with the greatest activity observed against *L. monocytogenes* ATCC 13932 and *Enterococcus faecalis* ATCC 7080 with a low MIC of 6.3 µg/mL. This was followed by the reference, laboratory, and clinical *S. aureus* strains, as well as the clinical *E. faecalis* S1 and *E. faecium* S1 strains at MIC of 12.5 µg/mL, while W2-FL10 exhibited the lowest activity against *B. subtilis* ATCC 6051 (MIC of 31.3 µg/mL; Table 1). No activity was observed against the tested Gram-negative bacterial strains (indicated in the “Bacterial strains” section of Materials and Methods) (MIC > 125 µg/mL, results not shown). The inhibition concentration factor (IC_F_; describing the fold increase in the concentration of the peptide needed to progress from minimum and maximum inhibition), according to Rautenbach et al. (17), was additionally determined (Table 1). Membrane active antimicrobial peptides having cooperative interactions to form membrane pores, channels, and lesions exhibit IC_F_ values from >1 to 6, as was found for W2-FL10 (17). The activity of melittin was tested against the Gram-positive bacteria, which showed that this peptide was slightly more active than W2-FL10 against *S. aureus* ATCC 25923, *S. aureus* RN4220, MRSA Xen 30, *L. monocytogenes* ATCC 13932, and *E. faecalis* ATCC 7080 but was less active against *B. subtilis* ATCC 6051, *E. faecalis,* and *E. faecium* clinical strains (Table 1). To explore the mode of action of W2-FL10 against strains from different sources (reference, clinical, and laboratory), *S. aureus* ATCC 25923, *S. aureus* RN4220, and MRSA Xen 30 were selected as representative Gram-positive bacteria for further studies.

**TABLE 1:** Summary of activity parameters of W2-FL10, in comparison with MIC values of melittin, against a panel of Gram-positive bacteria^*h*^

		W2-FL10					Melittin
Target cell	Source of strain/cells	^*a*^MIC μg/mL (μM)	^*b*^IC_50_ ± SD µg/mL (n)	^*c*^IC_F_ (MIC/IC_50_)^2^		^*g*^Selectivity index	MIC μg/mL (μM)
*S. aureus* ATCC	Reference	12.5 (17.1)	5.5 ± 0.3 (3)	5.2		5.3	32 (11.2)
*S. aureus* RN4220	Laboratory	12.5 (17.1)	5.7 ± 0.6 (3)	4.9		5.1	32 (11.2)
*S. aureus* Xen 30 (MRSA Xen 30)^*d*^	Clinical	12.5 (17.1)	5.7 ± 0.6 (3)	4.8		5.1	32 (11.2)
*L. monocytogenes* ATCC 13932	Reference	6.3 (8.5)	3.8 ± 0.2 (3)	2.7		7.6	32 (11.2)
*B. subtilis* ATCC 6051	Reference	31.3 (42.7)	13.4 ± 0.8 (3)	3.8		2.2	>32 (>11.2)
*E. faecium* S1	Clinical	12.5 (17.1)	5.4 ± 0.1 (3)	5.9		5.4	>32 (>11.2)
*E. faecalis* ATCC 7080	Reference	6.3 (8.5)	3.2 ± 0.3 (3)	5.4		9.1	32 (11.2)
*E. faecalis* S1	Clinical	12.5 (17.1)	5.2 ± 0.5 (3)	5.4		5.6	>32 (>11.2)
Chinese hamster ovary cells	Reference	*36.6 (50.0)^e^*	*29.0 ± 5.9 (3)^f^*	*1.6*		ND^*i*^	ND

^*a*^ MIC, determined as concentration giving ≥90% growth inhibition according to OD measured at 600 nm.

^*b*^ IC_50_, 50% inhibition concentration determined from sigmoidal curve fit to dose response.

^*c*^ IC_F_, inhibition concentration factor as described by Rautenbach et al. (17).

^*d*^ MRSA, methicillin-resistant *S. aureus*.

^*e*^ MLC, minimum lethal concentration.

^*f*^ 50% lethal concentration (LC_50_) determined with an MTT viability assay.

^*g*^ Selectivity index = LC_50_/IC_50_.

^*h*^ Refer to Fig. S1 for the dose-response curves.

^*i*^ ND, not determined.

In addition to the antibacterial assays, a basic cytotoxicity assessment was performed using an 3-(4,5-dimethylthiazol-2-yl)-2,5-diphenyltetrazolium bromide (MTT) assay with a Chinese hamster ovarian (CHO) cell line to determine the selectivity of W2-FL10. In comparison to the control (emetine), which had an LC_50_ value of approximately 40 nM against the CHO cell line, W2-FL10 displayed low to moderate cytotoxicity with an LC_50_ of 40 µM (29.0 ± 5.9 µg/mL). The arbitrary selectivity index was 2 to 9 for the IC_50_ values of W2-FL10 recorded against the tested Gram-positive bacteria (Table 1).

### Effect of Mg^2+^, Ca^2+^, DOPG, and LTA on W2-FL10 activity

**Fig 2:**
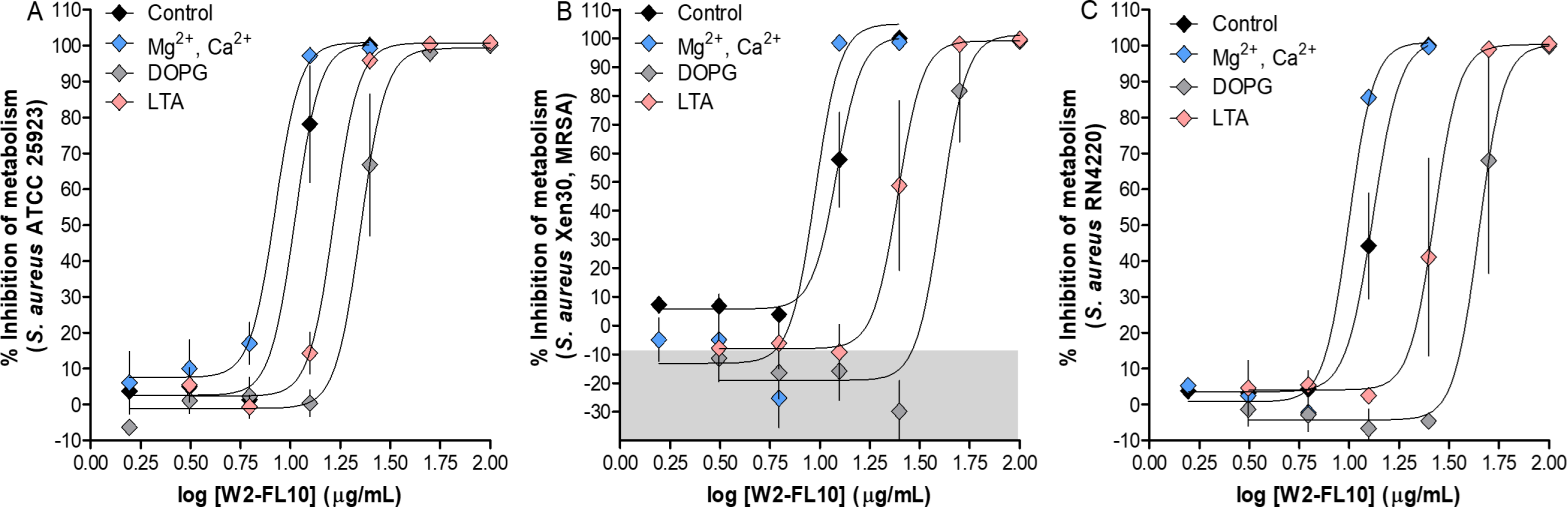
Influence of cations, DOPG, or LTA of *S. aureus* on the antibacterial activity of W2-FL10. Dose-response curves of W2-FL10 against *S. aureus* ATCC 25923 (**A**), MRSA Xen 30 (**B**), and *S. aureus* RN4220 (**C**) were determined using the resazurin broth microdilution assay in the presence of cations, DOPG, or LTA of *S. aureus*. The gray shaded area indicates cell stress. Data in A, B, and C are the mean of 3–4 determinations with standard error. The sigmodal fits all had R^2^ > 0.99 and were used to determine the inhibition parameters described in the text. Refer to Table S1 for the summary of inhibition parameters and Tables S2 and S3 for statistical analyses.

As cations can interact with certain lipopeptides and influence their potency, broth microdilution assays coupled with resazurin as vitality dye were used to investigate the influence of cations (Ca^2+^ and Mg^2+^) on the antibacterial potency of W2-FL10. In addition, to study the interaction of W2-FL10 with bacterial membrane lipids, commercial LTA from *S. aureus* and DOPG (all three at 1 mg/mL in 10% EtOH) were formulated with W2-FL10, as these lipids form part of the initial outer barrier of the lipopeptide and serve as potential targets of W2-FL10 (Fig. 2A through C; Table S1).

To determine the influence of cations on the potency of W2-FL10, two medium compositions namely Mueller Hinton broth (MHB) and cation adjusted (CA)-MHB were used. The dose-response inhibition parameters, observed with the metabolic dye resazurin, indicated a small but significant decrease (*P* < 0.05) in the IC_50_ value of W2-FL10 against *S. aureus* ATCC 25923, with an IC_50_ of 10.6 µg/mL in MHB reduced to 8.4 µg/mL in CA-MHB, combined with a decrease in IC_F_ from 4.3 to 2.2 (Fig. 2A; Tables S1 and S2). Similarly, a statistically significant reduction was observed for *S. aureus* RN4220 in the presence of Ca^2+^ and Mg^2+^, with IC_50_ value of 13.2 µg/mL in MHB reduced to 10 µg/mL in CA-MHB (*P* < 0.01), but this did not translate into a lower MIC or change in IC_F_ (Fig. 2C; Tables S1 and S2). MRSA Xen 30 did not exhibit a significant decrease in IC_50_, but a noteworthy decrease in the IC_F_ from 3.4 to 1.7. Overall, the presence of Mg^2+^ and Ca^2+^ influenced the potency of W2-FL10 against the *S. aureus* strains, *S. aureus* RN4220>ATCC25923>MRSA Xen 30, with small but significant increases in the antimicrobial activity observed. This change, specifically the IC_F_ change, could indicate that the divalent cations induced a change in the mode of action or promoted the active form of the lipopeptide by either binding to it and/or limiting inactive oligomer formation (18). Refer to Tables S2 and S3 for detailed statistical analysis of the influence of Ca^2+^ and Mg^2+^ on W2-FL10 activity.

The antibacterial potency of W2-FL10 in the presence of 100 µg/mL of commercial LTA from *S. aureus* was then determined to investigate whether this lipopeptide can interact and bind to this cell wall lipid. Results indicated a 1.6-fold increase (*P* < 0.001) in the IC_50_ of W2-FL10 against *S. aureus* ATCC 25923 in the presence of LTA*,* with the IC_50_ value of 10.6 µg/mL increased to 16.6 µg/mL (Fig. 2A; Tables S1 and S2). Similarly, a twofold increase (*P* < 0.001) in the IC_50_ of W2-FL10 was observed against MRSA Xen 30 in the presence of LTA, with an IC_50_ value increase from 12.2 μg/mL to 24.6 μg/mL (Fig. 2B; Tables S1 and S2). In addition, a twofold IC_50_ increase from 13.2 μg/mL to 26.8 μg/mL (*P* < 0.001) of W2-FL10 was observed against *S. aureus* RN4220 in the presence of LTA (Fig. 2C; Tables S1 and S2). The presence of LTA resulted in a shift in the dose-response curves of W2-FL10 without changing the IC_F_ values or increasing the IC_F_ (Table S1), indicating that the presence of LTA lowers the antibacterial potency of W2-FL10, without influencing the mode of action. LTA could therefore be one of the W2-FL10 targets in the cell wall, with free LTA competing for the lipopeptide, effectively lowering availability for interaction with the target cell wall. Refer to Tables S2 and S3 for detailed statistical analysis of the influence of LTA on W2-FL10 activity.

The antibacterial potency of W2-FL10 in the presence of 100 µg/mL of DOPG was also determined, to investigate if this lipopeptide can interact and bind to this major bacterial membrane lipid. A twofold increase (*P* < 0.001) in the IC_50_ value (from 10.6 to 22.5 µg/mL) of W2-FL10 was observed against *S. aureus* ATCC 25923 in the presence of DOPG (Fig. 2A; Tables S1 and S2). Similarly, a 3.3-fold increase (*P* < 0.001) in the IC_50_ of W2-FL10 was observed against MRSA Xen 30 in the presence of DOPG, with an IC_50_ value increase from 12.2 to 40.6 µg/mL (Fig. 2B; Tables S1 and S2). In addition, a 3.4-fold increase (*P* < 0.001) in the IC_50_ (from 13.2 to 44.6 µg/mL) of W2-FL10 was observed against *S. aureus* RN4220 in the presence of DOPG (Fig. 2C; Tables S1 and S2). The presence of DOPG thus significantly (*P* < 0.001) influenced the dose-response curves of W2-FL10 with variable changes in the IC_F_ values, namely an increase to 6.3 for *S. aureus* ATCC 25923 indicating DOPG competition, and a decrease to 2.4 and 2.2 for Xen30 MRSA and *S. aureus* SN4220, indicating a crucial threshold concentration was necessary for activity against these two strains. This implies that negative phosphatidylglycerol may be one of the W2-LF10 targets in the bacterial membrane, with the DOPG vesicle that will form in an aqueous medium competing for interaction with the amphipathic lipopeptides. Refer to Tables S2 and S3 for detailed statistical analysis of the influence of DOPG on W2-FL10 activity.

### Time-kill kinetics

Time-kill assays were performed for W2-FL10 against *S. aureus* ATCC 25923, *S. aureus* RN4220, and MRSA Xen 30 to determine the bacteriostatic (suppresses growth) or bactericidal (>3 log reduction in CFUs) activity of the compound, allowing for the determination of cell survival percentage per minute (19). The half-life (i.e., time taken to achieve 50% bactericidal activity) of an antibiotic can then be extrapolated from this data, to provide an indication of the kill rate.

**Fig 3:**
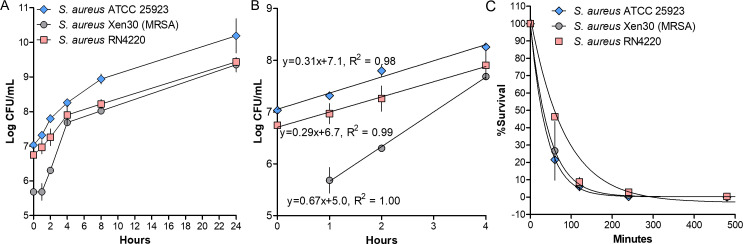
Bacterial growth and time-killing kinetics of *S. aureus* ATCC 25923, *S. aureus* RN4220, and MRSA Xen 30. Bacterial growth kinetics over 24 h was determined using plate counts (CFU/mL) (i.e., untreated control cultures of *S. aureus* strains in the time-kill assay) (**A**), growth in the first 4 h showing the logarithmic trends (i.e., log of the CFU/mL value determined using the untreated control cultures of *S. aureus* strains in the time-kill assay) (**B**), and exponential time-kill kinetics (% survival over time, determined with CFU/mL during the time-kill assay in comparison to the untreated control cultures) (**C**) when challenged at 25 µg/mL of W2-FL10. Data are the means of triplicate determinations with standard error.

When comparing the growth of the three *S*. *aureus* strains, it can be seen that MRSA Xen 30 and *S. aureus* RN4220 have a similar trend toward the stationary phase (Fig. 3A). Conversely, *S. aureus* ATCC 25923 and *S. aureus* RN4220 have similar growth rates, while MRSA Xen 30 has a faster initial growth rate (Fig. 3B). In comparison to the positive control (growth curves without antimicrobial treatment) as indicated in Fig. 3A, percentage survival significantly decreased over time after exposure to W2-FL10 (Fig. 3C). The bactericidal half-life of W2-FL10 was reached against *S. aureus* ATCC 25923 at only 28 ± 4 min after a challenge with 25 µg/mL of W2-FL10 (Fig. 3C), while at 12.5 µg/mL of W2-FL10, the resulting half-life was 47 ± 8 min (results not shown). A similar half-life was observed against MRSA Xen 30 at 31 min after dosing with 25 µg/mL of W2-FL10, while a similar half-life of 48 ± 14 min was observed at 12.5 µg/mL of W2-FL10. The longest bactericidal half-life of 45 ± 1 min was observed against *S. aureus* RN4220 after exposure to 25 µg/mL of W2-FL10 (Fig. 3C), while at 12.5 µg/mL of W2-FL10, the half-life was 67 ± 3 min (results not shown). Overall, W2-FL10 is a fast-acting lipopeptide, with short half-lives observed against all three *S*. *aureus* strains. This rapid killing kinetics indicated a lethal effect on crucial cell functionality, such as membrane function. Such an effect can be via a membrane-active mode of action, which is supported by the antagonistic effect of DOPG on W2-FL110.

### Membrane depolarization

**Fig 4:**
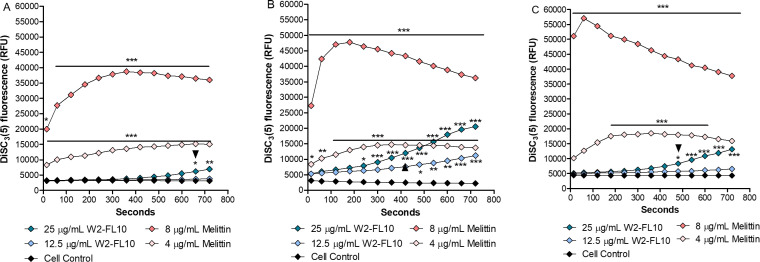
Membrane depolarization by melittin and W2-FL10. The DiSC_3_(5) fluorescence measured over 720 s, as a reflection of the membrane potential of *S. aureus* ATCC 25923 (**A**), MRSA Xen 30 (**B**), and *S. aureus* RN4220 (**C**) treated with W2-FL10 or melittin. The arrows indicate the time point when the doubling of fluorescence occurred at 25 µg/mL of W2-FL10. Statistical comparison between control (cells alone) and treatment with peptide was done using a two-way analysis of variance (ANOVA) with Bonferroni’s post hoc test. Significant difference is indicated as *P < 0.05, **P < 0.01, and ***P < 0.001, for *n* = 2 independent determinations.

To determine whether W2-FL10 causes membrane depolarization in the three *S*. *aureus* strains, the membrane potential was assessed by monitoring the release of the voltage-sensitive dye DiSC_3_(5) (Fig. 4A through C) (20). An initial delay in depolarization was observed against all three *S*. *aureus* strains after exposure to W2-FL10. A statistically significant increase in fluorescence occurred between ~250 and 720 s (~4–12 min) for exposure of all three *S*. *aureus* strains to 25 µg/mL of W2-FL10 (Fig. 4A through C). The fluorescent signal doubled against *S. aureus* ATCC 25923 (Fig. 4A), MRSA Xen 30 (Fig. 4B), *S. aureus* RN4220 (Fig. 4C) at 660 s (11 min), 420 s (7 min), and 480 s (8 min), respectively, when exposed to 25 µg/mL W2-FL10. After 720 s (12 min) of exposure to 25 µg/mL of W2-FL10, a 2.2-, 3.6-, and 3.8-fold increase in fluorescence was recorded for *S. aureus* ATCC 25923, *S. aureus* RN4220, and MRSA Xen 30, respectively. In contrast, the increase in fluorescent signal was negligible for the challenge at 12.5 µg/mL of W2-FL10 for two of the three *S. aureus* strains up to 720 s (12 min) (Fig. 4A and C). Only MRSA Xen 30 showed appreciable depolarization (Fig. 4C).

As depolarization control, melittin at 8 µg/mL, caused rapid depolarization within 60–320 s (1–5 min). Some differences were observed between the three strains at the different melittin concentrations, indicating that there may be differences in the cell wall composition allowing access, the negative charge of the membrane allowing membrane interaction with the cationic melittin and/or fluidity that can influence pore formation (21) (Fig. 4A through C). As expected, no increase in DiSC_3_(5) fluorescence was observed for the negative control of the three *S*. *aureus* strains (Fig. 4A through C).

### Membrane permeabilization

**Fig 5:**
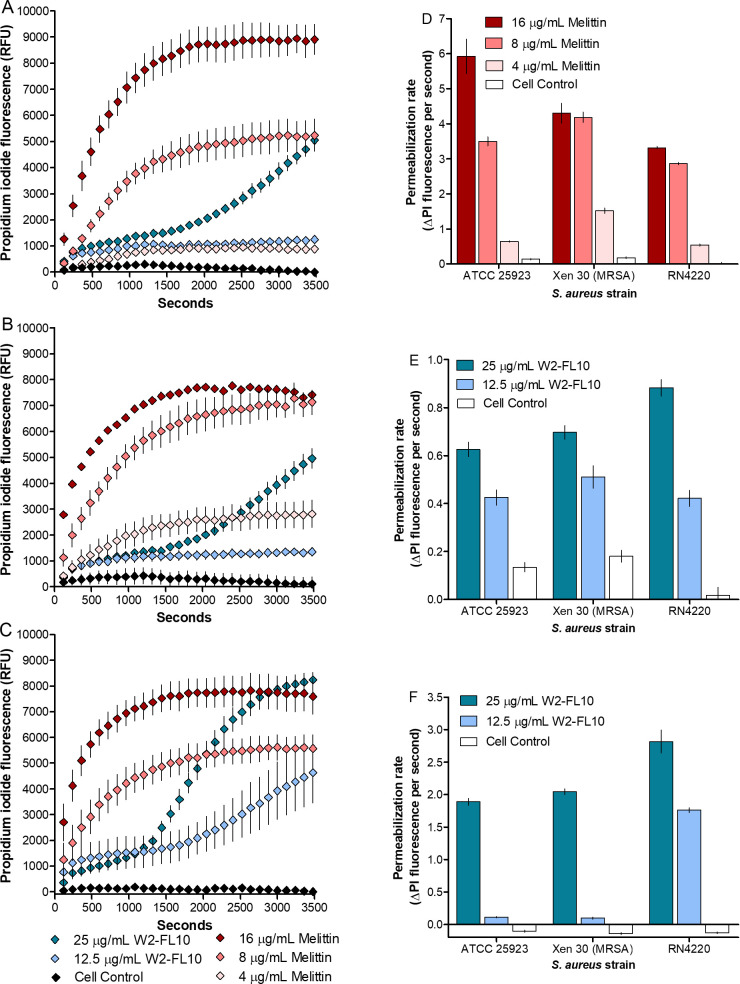
Membrane permeabilization by melittin and W2-FL10. The PI fluorescence measurement over 60 min, as a reflection of membrane permeability of (**A**) *S. aureus* ATCC 25923, (**B**) MRSA Xen 30, and *S. aureus* RN4220 (**C**) treated with W2-FL10 or melittin. Comparison of the PI fluorescence uptake rates of *S. aureus* ATCC 25923, MRSA Xen 30, and *S. aureus* RN 4220 when treated with melittin from 6 to 16 min (360–960 s) (**D**), W2-FL10 from 6 to 18 min (360–1080 s) (**E**), and W2-FL10 from 34 to 50 min (2,040–3,000 s) (**F**). Data points are the mean of 3 to 4 independent determinations with standard error in A–C and standard deviation in D–F. In B and D, the 16 µg/mL melittin data set is the mean of duplicate determinations. Refer to Table S4 for statistical analyses.

To determine whether W2-FL10 causes membrane permeability by forming lesions or pores within the cell membranes of the three *S*. *aureus* strains, the influx of propidium iodide (PI) over compromised membranes was monitored. The intracellular PI interacts with nucleic acids within the cell, resulting in a fluorescent signal (22). Fluorescence was measured over 1 h after challenge with 12.5 or 25 µg/mL of W2-FL10 (Fig. 5A through C).

An initial slow rate of permeabilization up to 18 min (1,080 s) was observed against all three *S*. *aureus* strains following treatment with W2-FL10 (Fig. 5A through F). The rate of PI uptake over this period was between 0.3 and 0.6 fluorescent units/s (FU/s), which was about 10-fold lower than that of 4 and 8 µg/mL melittin (Fig. 5D and E). These results correlated with the difference in membrane depolarization seen between the overt lytic peptide melittin and W2-FL10 over 720 s (12 min) (refer to Fig. 4). Over the first 18 min, *S. aureus* ATCC 25923 (Fig. 5A and E) and MRSA Xen 30 (Fig. 5B and E) showed similar kinetics after a 25 µg/mL of W2-FL10 challenge, but both differed significantly (*P* < 0.001) from that of *S. aureus* RN4220 (Fig. 5C and B) (refer to Table S4 for statistical analysis). A substantial increase in the PI uptake rate occurred only after 18 min W2-FL10 exposure, approaching that of melittin in the first 18 min, for the three *S*. *aureus* strains challenged with 25 µg/mL of W2-FL10 (Fig. 5A through C, E, and F). A 50% PI uptake was observed for the *S. aureus* ATCC 25923 and MRSA Xen 30 strains from 2,280 to 2,400 s (38–40 min) (Fig. 5A and B). In comparison, *S. aureus* RN4220 was more sensitive with a 50% PI fluorescence increase from 1,680 to 1,800 s (12–30 min) (Fig. 5C). A ≥90% uptake in fluorescence was observed within 3,360 s (56 min) for *S. aureus* ATCC 25923, 3,240 s for MRSA Xen 30, and 2,760 s (46 min) for *S. aureus* RN4220 (Fig. 5A through C). However, this delayed sigmoidal trend in the permeabilization kinetics, after exposure to 25 µg/mL of W2-FL10, is very different from that observed for melittin with well-known non-specific lytic activity (23). Melittin challenge at 8 µg/mL caused rapid membrane permeabilization for all three *S*. *aureus* strains with an exponential increase in PI fluorescence signal and 50% PI uptake within 120–240 s (2–4 min) (Fig. 5A through C). This difference in the permeabilization and depolarization trends indicated that W2-FL10 acted differently on bacterial membranes.

### Scanning electron microscopy

**Fig 6:**
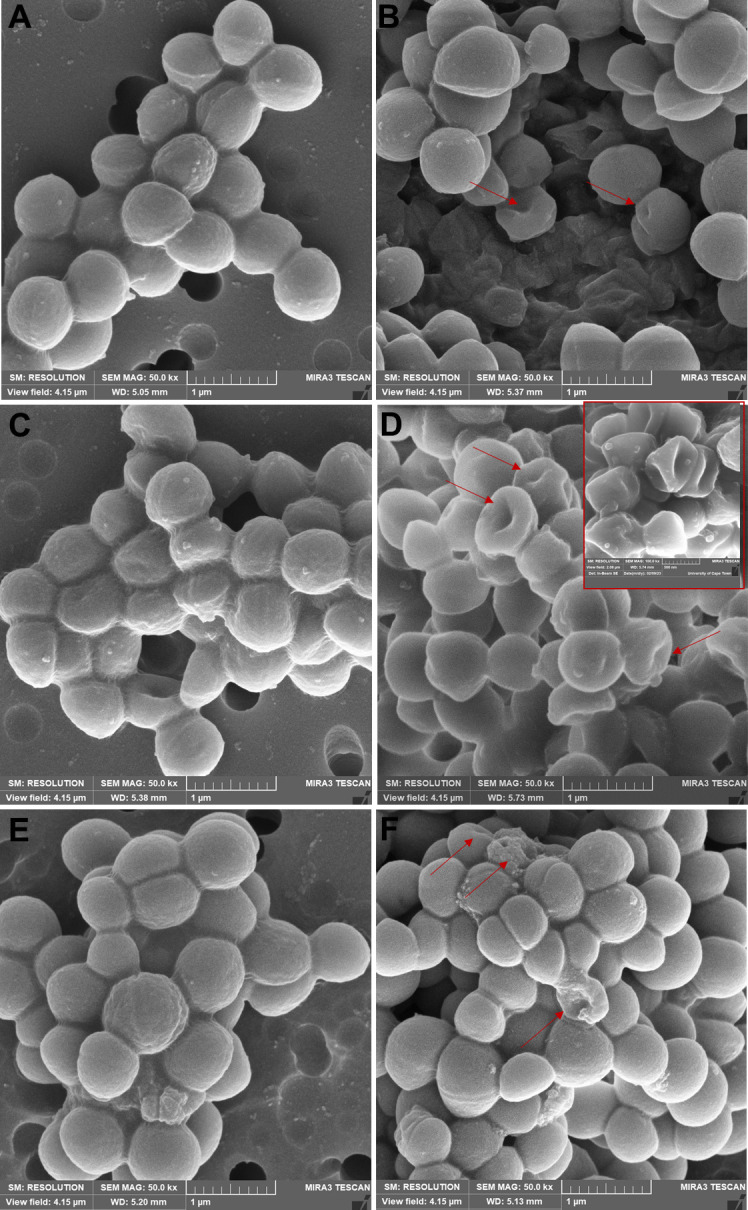
SEM images (1 µm) of *S. aureus* ATCC 25923 (**A and B**), *S. aureus* RN4220 (**C and D**), and MRSA Xen 30 (**E and F**) before (left panel) and after treatment with 25 µg/mL of W2-FL10 for 1 h (right panel). An increased magnification (500 nm) of damaged *S. aureus* RN4220 cells is indicated in the red block of Fig. 6D.

The effects of W2-FL10 on the morphology of the three *S*. *aureus* strains were investigated using SEM. The SEM images were captured before and after 1 h treatment with 25 µg/mL of W2-FL10. Figure 6 (left panel) shows the control *S. aureus* strains that have the expected spherical shape and no cell damage. Minor morphological changes were then observed for *S. aureus* ATCC 25923 (Fig. 6A and B) and MRSA (Fig. 6E and F) after exposure to W2-FL10, while more significant cell damage [formation of primarily one large lesion per cell was observed in the cell capsule (indicated by red arrows)] was observed against *S. aureus* RN4220 (corresponding to the PI leakage for this strain) (Fig. 6C and D).

## DISCUSSION

Key parameters contributing to the antimicrobial activity of lipopeptides include hydrophobicity and net charge (5, 24). Serrawettin W2-FL10 is a relatively small and moderately amphipathic lipopeptide with a neutral charge. This lipopeptide displayed potent activity against eight of the nine Gram-positive bacteria included in the test panel, with *B. subtilis* ATCC 6051 exhibiting some resistance, possibly due to the production of a surfactin complex containing anionic lipopeptides (25), which has been shown to antagonize activity by binding to the potent antimicrobial peptide gramicidin S (11, 26). W2-FL10 exhibited nearly identical activity, in terms of the IC_50_, MIC, and IC_F_ parameters, against five of the nine bacteria in the test panel, indicating similar bacterial target(s), concentration(s), and mode of action. Conversely, W2-FL10 exhibited its highest activity, outperforming that of melittin, against the *L. monocytogenes* ATCC 13932 and *E. faecalis* ATCC 7080 reference strains, with lower IC_F_ values of 2.7 and 5.4, respectively. This correlated with the IC_F_ value of the frog peptide, PGla reported at 2.5, and the lytic cyclic decapeptide gramicidin S at 5.7 against *Escherichia coli* (27). Such low IC_F_ values are indicative of potent cooperative activity (17), that could include self-promoted peptide uptake into the membrane, as observed for PGla (27) and/or self-promoted uptake into the cytosol. The low IC_F_ and MIC values of W2-FL10 (Table 1) also correlated well with that of the cyclodecapeptide tyrocidine C against *L. monocytogenes* B73 at 1.9 and 13 µM, respectively (28). Tyrocidine C forms dimers and self-assembles (29), similar to what was observed for W2-FL10 (refer to Fig. 1C). Tyrocidine C was shown to form ion-conducting pores with pore formation dependent on self-assembly and cooperative interactions, as well as having other membrane effects and possible intracellular targets (30).

The initial barrier for W2-FL10 when targeting Gram-positive bacteria is the LTA cell wall polymer (31) and peptidoglycan layer. LTA is a major constituent of the cell wall of Gram-positive bacteria and the structure of LTA is species-specific (32). In particular, the LTA of *S. aureus* is an amphipathic molecule comprised of a lipid anchor (linked to the cytoplasmic membrane) and a negatively charged hydrophilic glycerophosphate backbone protruding through the thick peptidoglycan layer, thus exposed to the surrounding environment (33, 34). Results in this study indicated that the addition of the LTA of *S. aureus* to the antibacterial assay increased the IC_50_ of W2-FL10 about twofold against the three selected *S. aureus* strains, indicating that W2-FL10 may interact with the LTA. Generally, cationic lipopeptides and antimicrobial peptides, such as melittin interact with LTA and peptidoglycan through salt-bridges and other electrostatic interactions (20). These interactions weaken the cell wall, which facilitates penetration through the thick peptidoglycan layer, allowing the lipopeptide/peptide to interact with the bacterial membrane (20, 35, 36). As the lipopeptide in this study is non-ionic, only non-ionic interactions between LTA and W2-FL10 are expected to occur. In our assay, where LTA is not anchored in its natural membrane environment, it is probable that W2-FL10 was also interacting with the hydrophobic lipid tail of the free LTA. However, further investigation into the role of LTA and peptidoglycan in the initial interactions of serrawettins with Gram-positive target cells is required, as the mode of traversing the cell wall of the serrawettins and other non-ionic lipopeptides has not been elucidated.

Once amphipathic peptides like W2-FL10 reach the bacterial cell membrane, they interact with and bind to the phospholipid bilayer (10, 31, 35). In *S. aureus*, a major component of the phospholipid bilayer is phosphatidylglycerol (37). To investigate the interaction between W2-FL10 and phosphatidylglycerol, DOPG was incorporated into the antibacterial assay. Results indicated that the addition of the negatively charged DOPG to W2-FL10 significantly increased its IC_50_ by two to fourfold against the three *S*. *aureus* strains. As the activity was lost, W2-FL10 may have interacted with the DOPG vesicles that naturally form in the aqueous medium and would therefore also bind to these negative phospholipids in the cytoplasmic membrane. This phospholipid interaction is generally based on electrostatic and hydrophobic interactions, where lipopeptides tend to insert their hydrophobic fatty acid moiety into the phospholipid bilayer (interacting with the hydrophobic lipids), while the hydrophilic moiety points toward the hydrophilic head-groups of the phospholipids (5, 31). For W2-FL10, the hydrophobic fatty acid chain in the lipopeptide structure is flanked by three hydrophobic amino acids (i.e., two Leu and one Phe), while the hydrophilic amino acids are positioned closely together on the other side of the structure. It is hypothesized that the hydrophilic moiety of the peptide interacts with the polar glycerol and anionic phosphate in the lipid headgroups in the cell membrane. For the non-ionic W2-FL10, this may be a weaker interaction due to the lack of ionic interactions. Accordingly, the interaction of the lipopeptide with Ca^2+^ and Mg^2+^ could lead to ionic interactions and possibly improved activity of W2-FL10. This improved interaction could explain both the lower MIC and much improved IC_F_ of nearly two for two of the *S. aureus* targets. For example, mode of action studies with model membranes have revealed that the anionic lipopeptide, daptomycin, complexes with Ca^2+^ and only interacts with liposomes when phosphatidylglycerol is present (38–40). After initial headgroup interaction, the hydrophobic moiety of W2-FL10 (fatty acid and three amino acids) is possibly incorporated into the bilayer due to a strong hydrophobic driving force, while the hydrophilic amino acids may remain associated with phospholipid head groups. Moreover, Gram-negative bacteria have significantly lower phosphatidylglycerol within their membranes (41), which may also result in reduced activity.

Following the insertion of the hydrophobic moiety of a lipopeptide into the bilayer, lipopeptides tend to oligomerize in the membrane (5, 31). As shown in this study (refer to Fig. 1C) and by Clements-Decker et al. (6) using MS, W2-FL10 could form stable, non-covalent dimers and higher oligomers. It is probable that oligomerization is occurring within the bacterial membrane, and may result in transmembrane pores/lesions, similar to that of daptomycin (5). Although further analysis is required to confirm transmembrane oligomerization, a membrane depolarization assay using the voltage-sensitive dye, DiSC_3_(5), was conducted to confirm the initial disruption of membrane potential (formation of ion-specific channels) (42). W2-FL10 disrupted the membrane potential of the three *S*. *aureus* strains within 720 s at high concentrations, indicating that perforation of the membrane occurred and allowed for the leakage of small ions. It is important to note the initial delay in depolarization in comparison to melittin, suggesting that the formation of lesions (such as ion-specific pores or channels) is time-dependent on a self-assembly step to form oligomeric pores/channels, which is similarly observed with stephensiolides produced by *Serratia* species (43). Bacteria can cope with some of the ion leakage by upregulating the membrane pumps (44) which could overcome depolarization. However, if the self-assembly process of the peptides does not lead to a specific pore or channel size and is far from equilibrium, it could cause enlarging of the lesions over time. This will allow for the leakage of larger molecules and nullify the bacterium’s response to stabilize the membrane potential. Therefore, membrane permeability was assessed by monitoring the influx of the membrane-impenetrable dye, PI (668 Da). This tracer molecule is relatively large and close to the size of the W2-FL10 (731 Da). Similar to the membrane potential dissipation, the membrane permeabilization of PI into *S. aureus* was delayed, compared to that of melittin, suggesting W2-FL10 has a different mechanism of action. It is likely that the larger lesion formation by W2-FL10 is time-dependent and ultimately led to the delayed leakage of PI, within 30–45 min. Following membrane depolarization and permeability, the W2-FL10 was found to have a correlating rapid kill rate. The half-life (indicating 50% cell death) was observed within 28 to 47 min after exposure to 25 µg/mL of W2-FL10, corresponding to the increase in permeability during this time frame. The fatal cell damage was confirmed by SEM analysis, where the formation of lesions and cell damage of *S. aureus* RN4220 was visible after 1 h of exposure to W2-FL10.

When comparing the membrane depolarization, permeabilization, and time-kill kinetics results of W2-FL10, obvious differences were observed against the three *S*. *aureus* strains (refer to Fig. 3 through 5), despite nearly identical inhibition parameters (refer to Table 1). For the depolarization analysis, MRSA Xen 30 was the most sensitive, while for the membrane permeabilization analysis, *S. aureus* RN4220 was the most sensitive. The formation of pores/channels and their progression into larger lesions is dependent on the lipopeptide interaction with the phospholipids and its oligomerization (self-assembly) within the membrane. The phospholipid composition of the cell membrane of *S. aureus* has been shown to vary between strains (37), potentially influencing the fluidity and charge of the cell membrane. It is thus hypothesized that this variation in membrane charge may have affected the interactions of the *S. aureus* strains with W2-FL10, while changes in fluidity may have influenced the formation of stable pores or larger lesions via oligomerization of W2-FL10. When considering the time-kill kinetics, *S. aureus* ATCC 25923 and MRSA Xen 30 showed similar, but faster kinetics than *S. aureus* RN4220, which did not correlate with either depolarization or permeabilization trends. Furthermore, if one considers that >90% bactericidal action occurred within 4–8 h at MIC, a concentration where both depolarization and permeabilization were low, more than one sensitive target needs to be considered. Notably, melittin at 8 µg/mL caused membrane damage. At higher W2-FL10 concentrations the membrane would be a major target; however, it could be that the low membrane permeabilization is part of self-promoted uptake in an intracellular mode of action. Finding such alternative target(s) is complex and will be considered in future studies.

Despite W2-FL10 showing promise as an antibiotic candidate, the higher hydrophobicity of this molecule may lead to inherent drawbacks. Some lipopeptides behave as non-cell-selective antimicrobials due to their higher hydrophobicity, leading to increased toxicity (24, 31). Our analyses indicated that W2-FL10 exhibited low to moderate cytotoxicity in comparison to the control, emetine, and was within the optimal lipophilicity range for systemic application. Although poor solubility was observed, this result may elucidate the improved activity detected in the presence of cations during the antimicrobial assay, as the presence of Ca^2+^ and Mg^2+^ may have improved the solubility. Moreover, the development of resistance is an important limitation in the therapeutic application of certain antibiotics. Although not monitored in this study, reports have indicated that *S. aureus* and *E. faecalis* have begun to display resistance to daptomycin (45). It is known that daptomycin-resistant strains of *S. aureus* or *E. faecalis* convert the negatively charged PG to its positively charged derivative alanyl- or lysyl-PG in the cell membrane, thereby reducing the affinity of daptomycin to the membrane and subsequent potency of the antibiotic (46). The non-ionic nature of W2-FL10 suggests that this mechanism of resistance will not influence the lifespan of W2-FL10. Combined with the possibility of both membrane and intracellular targets, the development of resistance of W2-FL10 will be less likely to occur. Moreover, due to the reduced size of the W2-FL10 structure (five amino acids and a C_10_ fatty acid chain) in comparison to daptomycin (13 amino acids and a C_10_ fatty acid chain) (45), substantial reductions in the manufacturing costs of W2-FL10 makes this lipopeptide appealing for therapeutic applications.

## MATERIALS AND METHODS

### Materials

Luria-Bertani (LB) agar and Tryptic soy agar (TSA) were obtained from Merck (Johannesburg, South Africa), while Tryptic soy broth (TSB), MHB, and CA-MHB (containing ~0.5 mM of Ca^2+^ and ~0.4 mM of Mg^2+^) were purchased from Sigma-Aldrich (St. Louis, MO, USA). Peptone glycerol (PG, pH 7.2 ± 0.2) broth is composed of 5 g peptone powder (Merck) and 10 mL glycerol (Promega, Wisconsin, USA). Melittin (>85% pure) was purchased from Merck. The HPLC-grade acetonitrile (MeCN) was purchased from Romil (Darmstadt, Germany). Clear (No. 655161) and black (No. 655086) Greiner CELLSTAR 96-well plates were purchased from Merck. White, Cliniplate 96-well microtiter plates were purchased from Thermo Fisher Scientific (Finland). DOPG was purchased from Avanti Polar lipids (Alabaster, AL, USA), while LTA from *S. aureus* was purchased from Sigma-Aldrich. Stock solutions of DOPG and LTA were prepared in 100% high-grade EtOH to a concentration of 10 mg/mL and aliquots were prepared in analytical grade H_2_O (prepared through a Millipore water filtration system) to 1 mg/mL. PI (Sigma-Aldrich) was prepared in analytical grade H_2_O to 1 mg/mL. Resazurin (Sigma-Aldrich) was prepared as a stock solution of 0.3 mg/mL in phosphate-buffered saline (PBS) and filter sterilized through a 0.22 µm filter. The 3,3'-dipropylthiadicarbocyanine iodide [DiSC_3_(5); Merck] was prepared in 100% dimethyl sulfoxide (DMSO; Sigma-Aldrich) as a stock concentration of 400 µM. BacTiter-Glo Microbial cell viability assay kit was purchased from Promega. The HEPES buffer was purchased from BioShop (Burlington, Canada). All lipid and antibiotic stock solutions were stored at −20°C.

### Bacterial strains

The *S. marcescens* NP2 strain was previously isolated from a wastewater treatment plant sample and the identity of the strain was confirmed via molecular typing (47). The *S. marcescens* NP2 strain was deposited in the South African Rhizobium Culture Collection (SARCC no. 3157). The test bacterial strains used in the broth microdilution assay included *L. monocytogenes* ATCC 13932, *E. faecalis* ATCC 7080, *S. aureus* ATCC 25923, *S. aureus* RN4220, MRSA Xen 30, *E. faecalis* S1, *E. faecium* S1*, B. subtilis* ATCC 6051, *E. coli* ATCC 417371, *P. aeruginosa* ATCC 27853, *K. pneumoniae* ATCC 13383, *A. baumannii* ATCC 19606, and *S. typhimurium* ATCC 14028. The test microorganisms and *S. marcescens* NP2 are curated and accessible in the Water Resource Laboratory culture collection in the Department of Microbiology at Stellenbosch University (SU). The bacterial strains were streaked from glycerol stocks onto LB agar, except the *Listeria* and *Enterococcus* strains, which were streaked onto TSA. All plates were incubated at 37°C for 18 to 24 h.

### Production and purification of lipopeptides

The production and purification of W2-FL10 were performed as described by Clements-Decker et al. (6). Briefly, *S. marcescens* NP2 was grown in peptone glycerol broth (500 mL; in triplicate) for 120 h at 30°C on an orbital shaker (MRCLAB, London, United Kingdom) set to 120 rpm. The NP2 broth cultures were centrifuged at 10,000 rpm for 20 min at 4°C and the cell-free supernatants were lyophilized. Thereafter, solvent extractions were performed using 70% MeCN in analytical quality H_2_O (vol/vol). The crude extracts were combined, lyophilized, analytically weighed, and used for RP-HPLC at LCMS Central Analytical Facility Unit (CAF, Stellenbosch University), as described by Clements-Decker et al. (6). Following purification, the W2-FL10 fraction was subjected to UPLC-HRMS^E^ to confirm the structure and determine the purity of the fraction, as described by Clements-Decker et al. (6). Purified W2-FL10 was analytically weighed to six digits to obtain an exact weight of the fraction and was re-weighed prior to the independent duplicate experiments of each assay.

### Lipophilicity and solubility

The lipophilicity and aqueous solubility (pH 7.4) of W2-FL10 were determined by the Drug Discovery and Development Centre at the University of Cape Town. Solubility was measured using a miniaturized shake-flask method in a 96-well plate (48, 49). Briefly, W2-FL10 was prepared to a 10 mM stock solution in 100% DMSO and 4 µL was added to a 96-well pate and evaporated using a GeneVac system. Phosphate buffer at pH 6.5 was then added to the wells and the plate was incubated for 24 h at 25°C with shaking. After incubation, the samples were centrifuged at 3,500 *× g* for 15 min and then transferred to an analysis plate. A calibration curve in DMSO for each sample between 10 and 220 µM was prepared and included in the analysis plate. Analysis was then performed by Agilent 1200 rapid resolution HPLC coupled to a Diode Array Detector (HPLC-DAD) and the solubility of each sample was determined from the corresponding calibration curve.

The lipophilicity of the compound was measured using a miniaturized shake-flask method, in 96-well plate format (48). Briefly, equal volumes of 1-octanol and phosphate buffer at pH 7.4 were added to each compound in a deep well plate. The plate was shaken vigorously for 2 h at 25°C. The phases were then carefully separated and transferred to another plate. Analysis was performed by HPLC-UV and log D values were determined from the peak areas of the compound in octanol and buffer phases.

### Antibacterial activity: broth microdilution assay

Serrawettin W2-FL10 (95% pure) was tested for antibacterial activity using a broth microdilution susceptibility assay (6), with melittin included as an antibiotic control. The serrawettin W2-FL10 was prepared in 70% MeCN to 1.00 mg/mL, from which aliquots were prepared using 70% MeCN to a concentration of 50 µg/mL or 500 µg/mL, while aliquots of melittin were prepared using analytical quality H_2_O to 128 µg/mL. Fifty microliters of the respective W2-FL10 aliquots or melittin were dispensed into a clear 96-well plate and a microdilution (in 70% MeCN for W2-FL10 or analytical quality H_2_O for melittin) was performed within the plate. The plate was air-dried to remove the solvent and placed in a desiccator with chloroform for 20 min for sterilization.

All the cultures (inoculated into 5 mL MHB, or 5 mL TSB for *Listeria* and *Enterococcus* strains) were incubated at 37°C to reach an optical density (OD) of 0.4 at 600 nm (∼10^7^ CFU/mL). A 1:20 dilution of the test strain (final concentration of ∼10^5^ CFU/mL) was prepared and 100 µL was dispensed into each well (final concentration of W2-FL10 at 1.5–125 µg/mL and melittin at 2–32 µg/mL). Sterile broth and the OD-adjusted inoculum were included as positive controls, while sterile broth (no inoculum) was included as a negative control. The 96-well plate was incubated for 18 h at 37°C. All the tests were performed in triplicate, with independent duplicates. Following incubation, the absorbance was measured using a microtiter plate reader at 600 nm. The 50% growth inhibition concentration (IC_50_) was determined using GraphPad Prism version 5 for Windows [GraphPad Software, San Diego, USA (www.graphpad.com)]. A sigmodal curve with variable slope was fitted to each set of dose-repose data according to Rautenbach et al. (17) using equation (1):


(1) $$Y=\frac{bottom+\left(top-bottom\right)}{1+10^{logIC_{50}\times Activity\;slope}}$$

The “top” and “bottom” are the maximum and minimum inhibition responses, respectively, for a particular set of dose-response data, while the activity slope is related to the Hill slope of a sigmoidal binding curve (16). Only sigmodal fits with R^2^ ≥ 0.95 were considered for the calculation of an IC_50_. The MIC value was determined for each set of dose-response data as the lowest concentration, resulting in ≥90% inhibition of the target cell growth. In addition to the MIC and IC_50_ inhibition parameters, an inhibition concentration factor (IC_F_), was calculated as outlined in Rautenbach et al. (17). The IC_F_ is indicative of the concentration increase from the highest concentration where no activity is observed to the MIC of a compound.

### Mammalian cell cytotoxicity assay

To determine the biocompatibility of W2-FL10 as a potential therapeutic agent, the *in vitro* cytotoxicity of W2-FL10 against the CHO cell line (50) was determined over 48 h by the Drug Discovery and Development Centre at the University of Cape Town, as previously described by Mosmann (51). Briefly, W2-FL10 was prepared to a 10 mM stock solution in 100% DMSO and dilutions were prepared in growth media. The CHO cell line density of 10^5^ cells/well in 96-well plates and allowed to attach for 24 h. Thereafter, the compound was added to the 96-well plates at various concentrations from 50 µM down to 16 nM and incubated for a further 48 h. At 44 h, MTT was added to the wells, and the plates were read 4 h later at 540 nm on a spectrophotometer. Emetine was included as the reference drug, as it shows non-specific cytotoxicity to mammalian cells. Finally, cell inhibition was plotted against concentration and the 50% lethal concentration (LC_50_) parameter and a minimum lethal concentration (MLC_90_) were obtained as described for the dose responses against bacterial targets above (equation 1).

### Influence of Mg^2+^, Ca^2+^, DOPG, and LTA on W2-FL10 antimicrobial activity

Based on the broth microdilution assay results for W2-FL10 and melittin, the *S. aureus* ATCC 25923 (reference), *S. aureus* RN4220 (laboratory; MSSA), or methicillin-resistant *S. aureus* Xen 30 (clinical; MRSA) strains were selected for further antibacterial studies. Resazurin (also referred to as Alamar Blue) is a blue, non-fluorescent dye that can be reduced to resorufin (a pink, fluorescent compound) by metabolically active cells. The dye can thus be used as a quantitative indicator of cell viability (52). Therefore, a broth microdilution assay coupled with resazurin [prepared to a stock solution of 0.3 mg/mL in PBS; (53)] was used to determine the influence of cations (Mg^2+^ and Ca^2+^), commercial LTA of *S. aureus* and DOPG on the potency of W2-FL10 against *S. aureus* ATCC 25923, *S. aureus* RN4220 and MRSA Xen 30 as described by Wu et al. (54).

For investigating the influence of cations on the antibacterial potency of W2-FL10, a clear 96-well plate was prepared with W2-FL10 and air dried as described in the “Antibacterial activity: broth microdilution assay” section. The *S. aureus* strains were inoculated into MHB or CA-MHB, grown to mid-log phase (OD_600_ of 0.5–0.6), and diluted to OD_600_ of 0.4. A 1:20 dilution of the test strain (final concentration of ∼10^5^ CFU/mL) was prepared and 100 µL was dispensed into each well (final concentration of W2-FL10 at 1.56 to 25 µg/mL). Sterile broth (i.e., MHB or CA-MHB) and the OD-adjusted inoculum were included in the assay as a positive control, while sterile broth (i.e., MHB or CA-MHB) was included as a sterility control. The plate was incubated for 24 h at 37°C.

Similarly, the possible interaction and binding of LTA of *S. aureus* and DOPG (both at 1 mg/mL in 10% EtOH) with the lipopeptide W2-FL10 was investigated (54). Briefly, a clear 96-well plate was prepared with W2-FL10 and air dried as described in the “Antibacterial activity: broth microdilution assay” section. Thereafter, 10 µL of LTA from *S. aureus* or DOPG was dispensed into the respective wells. The *S. aureus* strains were grown to mid-log phase in MHB (OD_600_ of 0.5–0.6) and diluted to OD_600_ of 0.4. A 1:20 dilution of test strain (final concentration of ∼10^5^ CFU/mL) was prepared, 100 µL was dispensed into each well (final concentration of W2-FL10 at 3.13 to 100 µg/mL), and the plate was incubated for 24 h at 37°C. Sterile broth, the OD-adjusted inoculum, and the respective LTA or DOPG were included in the assay as a positive control, while sterile broth and LTA or DOPG were included as a sterility control.

After incubation of both plates, 10 µL of resazurin (0.3 mg/mL) was added to each well and the plate was incubated at 37°C for 1 h. Fluorometric readings of the experimental plates were conducted using a Tecan Spark 10M Multimode Microplate Reader at 560 nm excitation wavelength and 590 nm emission wavelength. For all resazurin assays, the percentage inhibition was calculated as described by van Rensburg et al. (53). All experiments were performed in triplicate, with independent biological duplicates. The MIC was considered the concentration that resulted in ≥90% inhibition of growth based on the fluorometric readings. Dose-response curves (nonlinear regression using equation 1) were processed as described by Rautenbach et al. (17) (also see description above), and IC_50_ values were deduced from the best-fit sigmoidal curves (R^2^ ≥ 0.98). Statistical analyses were done by comparing W2-FL10 IC_50_, in the presence of Ca^2+^ and Mg^2+^, in the presence of DOPG, and in the presence of LTA toward the control condition each of the three *S*. *aureus* strains using one-way analysis of variance (ANOVA) with Bonferroni’s post hoc test.

### Time-kill kinetics

To determine the kill rate of the lipopeptide, exponentially growing *S. aureus* strains were prepared as described in the “Antibacterial activity: broth microdilution assay” section (OD_600_ = 0.4, ~10^7^ CFU/mL; 1:20 dilution was conducted to obtain ~10^5^ CFU/mL) (24, 55). Thereafter, 200 µL of the bacterial suspension (in MHB) was dispensed into a clear 96-well plate containing 12.5 or 25 µg/mL of W2-FL10, and plates were incubated at 37°C. Aliquots of 20 µL were collected at different time intervals (0, 1, 2, 4, 8, and 24 h), diluted in 180 µL PBS in a clear 96-well plate (from 10^−2^ to 10^−8^), and 100 µL of the respective dilution was spread onto LB agar plates. Undiluted samples of 20 µL were spot-plated onto LB agar to confirm the absence of cells at 8 and/or 24 h. The LB agar plates were then incubated overnight at 37°C and the CFU/mL was determined. A positive control of bacterial cells without peptide and a negative control with only LB were included. All the tests were performed in triplicate, with independent duplicates. The initial lytic rate (% lysis/min) was determined from the slope of the linear regression of % lysis versus time (up to 60 min) (27) and half-life was determined using the one-phase exponential decay equation, performed using GraphPad Prism 5 (GraphPad Software, San Diego, USA).

### Membrane depolarization assay

The membrane depolarization capability (ability to disrupt membrane potential) of the W2-FL10 against *S. aureus* ATCC 25923, *S. aureus* RN4220, or MRSA Xen 30 was determined using the voltage-sensitive fluorescent probe, DiSC_3_(5) (20). Briefly, black 96-well plates were prepared with W2-FL10 at 12.5 µg/mL and 25 µg/mL, while 4 and 8 µg/mL of melittin were used as a positive depolarization control, and the plates were air dried as described in the “Antibacterial activity: broth microdilution assay” section. The *S. aureus* strains were inoculated into MHB and grown to the mid-log phase (OD_600_ of 0.5–0.6). Cells were harvested by centrifugation at 3,000 rpm for 10 min and resuspended in HEPES buffer (5 mM, pH 7.4, containing 20 mM glucose; 100 mM KCl) to OD_600_ of 0.05. Thereafter, DiSC_3_(5) (stock concentration of 400 µM in 100% DMSO) was added to the cell suspension to a final concentration of 0.4 µM, and cells were incubated in the dark at room temperature for 40 min. Cell suspensions (100 µL) were then dispensed into the wells of the black 96-well plate. Sterile broth and the OD-adjusted inoculum with DiSC_3_(5) were included as a non-fluorescent control, sterile broth with DiSC_3_(5) was included as a sterility control, and melittin was included as membrane depolarization control. All the tests were performed in triplicate, with independent duplicates. Fluorometric readings of the plate were conducted at 1 min intervals over 12 min (readings began 60 s after cells were added to the plates) using a Tecan Spark at 622 nm excitation wavelength and 670 nm emission wavelength. Statistical comparison of the kinetic trends elicited by the treatment with peptide with the control (cells alone) was done using two-way ANOVA with Bonferroni’s post hoc test.

### Membrane permeability assay

The membrane permeability capabilities of the W2-FL10 against the *S. aureus* strains were determined using PI (56). Briefly, black 96-well plates were prepared with W2-FL10 at 6.25, 12.5, and 25 µg/mL, while 4–16 µg/mL of melittin was used as a positive permeability control, and the plates were dried as described in the “Antibacterial activity: broth microdilution assay” section. The *S. aureus* strains were inoculated into MHB, grown to mid-log phase (OD_600_ of 0.5–0.6), and diluted to OD_600_ of 0.3. PI (stock concentration of 1 mg/mL in analytical quality H_2_O) was added to the cell suspension to a final concentration of 10 µg/mL and cells were incubated in the dark at room temperature for 20 min. Thereafter, 100 µL of OD-adjusted PI cells in the mid-log phase were dispensed into each well. Sterile broth and the OD-adjusted inoculum with PI were included as a non-fluorescent control, while sterile broth with PI was included as a sterility control, and melittin was included as membrane depolarization control. All the tests were performed in triplicate, with independent duplicates. Fluorometric readings of the plate were conducted in 4-min intervals over 60 min (readings began ~60 s after cells were added to the plate) using a Tecan Spark at 535 nm excitation wavelength and 617 nm emission wavelength.

The leakage kinetics or PI permeabilization kinetic was assessed using a rate parameter of ΔPI fluorescence per minute. This was done by fitting linear regression lines on the linear sections, namely 6–16 min for melittin and 6–18 min, as well as 34–50 min for W2-FL10. The rates of PI permeabilization were compared between the three *S*. *aureus* strains using a one-way ANOVA with Bonferroni’s post hoc test.

### Scanning electron microscopy

SEM was performed to visualize the bacterial membrane morphology following treatment (24). Briefly, the *S. aureus* strains were prepared as described in the “Antibacterial activity: broth microdilution assay” section and harvested by centrifugation at 3,000 rpm for 10 min. The cells were re-suspended in PBS at a dilution of ~10^7^ CFU/mL (OD_600_ = 0.4). The diluted cultures were then treated with 25 µg/mL of W2-FL10, for 1 h, while cells without W2-FL10 were used as a control. Specimens were resuspended in 2.5% (vol/vol) glutaraldehyde solution in 0.1 M PBS (pH 7.2) and fixed for approximately 24 h. After the primary fixation, bacterial cells were pelleted and rinsed with PBS. Dehydration of samples through an ethanol series (30%, 50%, 70%, 90%, 95%, and 100% for 10 min each) was performed, followed by filtration onto a filter paper (pore size of 0.2 µm), which was glued onto an aluminum grid (11 mm diameter). The grid was dried with hexamethyldisilazane and coated with gold-palladium alloy. The grids were then inspected with a Tescan MIRA3 RISE Scanning Electron Microscope at the Electron Microscope Unit of the University of Cape Town.

## SUPPLEMENTAL MATERIAL

Supplemental tables and figure spectrum.02952-23-s0001.docx Tables S1-S4; Fig. S1.

ASM does not own the copyrights to Supplemental Material that may be linked to, or accessed through, an article. The authors have granted ASM a non-exclusive, world-wide license to publish the Supplemental Material files. Please contact the corresponding author directly for reuse.
