# Supplementary material for: Antibacterial efficacy and membrane mechanism of action of the Serratia-derived non-ionic lipopeptide, serrawettin W2-FL10
Source: Microbiol Spectr. 2024 Jun 6;12(7):e02952-23. doi: 10.1128/spectrum.02952-23 (PMC11218446; doi:10.1128/spectrum.02952-23)
Supplement: Supplemental tables and figure — Tables S1-S4; Fig. S1. [file spectrum.02952-23-s0001.docx]

**Antibacterial efficacy and membrane mechanism of action of the *Serratia*-derived non-ionic lipopeptide, serrawettin W2-FL10**

Tanya Decker^a^, Marina Rautenbach^b^, Sehaam Khan^a^ and Wesaal Khan^c*^

^a^Faculty of Health Sciences, University of Johannesburg, PO Box 17011, Doornfontein, 2028, South Africa

^b^BioPep^TM^ Peptide Group, Department of Biochemistry, Faculty of Science, Stellenbosch University, Private Bag X1, Stellenbosch, 7602, South Africa

^c^Department of Microbiology, Faculty of Science, Stellenbosch University, Private Bag X1, Stellenbosch, 7602, South Africa

*Corresponding Author - Wesaal Khan; Tel: +27 (21) 808 5804; E-mail: [wesaal@sun.ac.za](mailto:wesaal@sun.ac.za)

**Supplementary Data**

**Table S1** Summary of W2-FL10 metabolic inhibition parameters as determined with the resazurin assay (Van Rensburg et al. [1]), in the presence of Ca^2+^ and Mg^2+^. in the presence of DOPG and in the presence of LTA towards each of the three *S. aureus* strain. Note that parameters will differ from those determined in growth inhibition assays (refer to Table 1) which use different cell numbers, run over a much longer culturing period and use much less sensitive optical density measurements.

| ***S. aureus* strain**  **Challenge condition** | **MIC± SE (n)**  **μg/mL** | **IC_50_ ± SE (n)**  **μg/mL** | **IC_F_** |
| --- | --- | --- | --- |
| ***S. aureus* ATCC 25923**  Control  Ca^2+^ and Mg^2+^  DOPG  LTA | 21.9±3.1 (4) 12.5±0.0 (4) 56.3±15.7 (4) 41.7±8.3 (3) | 10.6±0.4 (4)  8.4±0.3 (4)  22.5±0.5 (4)  16.6±0.3 (4) | 4.3  2.2  6.3  6.3 |
| **MRSA Xen 30**  Control  Ca^2+^ and Mg^2+^  DOPG  LTA | 22.5±2.5 (5) 12.5±0.0 (4) 62.5±12.5 (4) 50.0±17.7 (3) | 12.2±0.2 (5)  9.5±1.5 (4)  40.6±2.0 (4)  24.6±0.1 (4) | 3.4  1.7  2.4  4.1 |
| ***S. aureus* RN4220**  Control  Ca^2+^ and Mg^2+^  DOPG  LTA | 25.0±0.0 (4) 25.0±0.0 (3) 66.7±16.7 (3) 50.0±0.0 (3) | 13.2±0.1 (4)  10.0±0.7 (3)  44.6±0.6 (3)  26.8±0.3 (3) | 3.6  6.3  2.2  3.5 |

SE: Standard error of the mean. n: number of experimental replicates.

**Table S2** Summary of statistical analyses of comparing W2-FL10 IC_50_’s (as given in Table S1) between the control condition, in the presence of Ca^2+^ and Mg^2+^, in the presence of DOPG and in the presence of LTA towards each of the three *S. aureus* strains using One-way Anova with Bonferroni’s post hoc test. The P-value indicates significance in row (number of experiments: n=3 to 5).

| ***S. aureus* strain**  **Condition** | **Ca^2+^ and Mg^2+^** | **DOPG** | **LTA** |
| --- | --- | --- | --- |
| ***S. aureus* ATCC 25923**  Control  Ca^2+^ and Mg^2+^  DOPG | <0.05  -  <0.001 | <0.001  <0.001  - | <0.001  <0.001  <0.001 |
| **MRSA Xen 30**  Control  Ca^2+^ and Mg^2+^  DOPG | ns  -  <0.001 | <0.001  <0.001  - | <0.001  <0.001  <0.001 |
| ***S. aureus* RN4220**  Control  Ca^2+^ and Mg^2+^  DOPG | <0.01  -  <0.001 | <0.001  <0.001  - | <0.001  <0.001  <0.001 |

**Table S3** Summary of statistical analyses of comparing W2-FL10 IC_50_’s towards the three *S. aureus* strains using One-way Anova with Bonferroni’s post hoc test. The P-value indicates row significance between the control condition, in the presence of Ca^2+^ and Mg^2+^. in the presence of DOPG and in the presence of LTA (number of experiments n=3 to 4).

| ***S. aureus* ATCC 25923**  Control  Ca^2+^ and Mg^2+^  DOPG  LTA | **MRSA Xen 30**  <0.01  ns  <0.001  <0.001 | ***S. aureus* RN4220**  <0.001  ns  <0.001  <0.001 |
| --- | --- | --- |
| **MRSA Xen 30**  Control  Ca^2+^ and Mg^2+^  DOPG  LTA | ***S. aureus* RN4220**  <0.05  ns  ns  <0.01 |  |

**Table S4** Summary of statistical analyses of comparing PI uptake rate upon 25 μg/mL W2-FL10 or 8 μg/mL melittin challenge of the three *S. aureus* strains using One-way Anova with Bonferroni’s Post Test. The P-value indicates row significance (number of experiments: n=3 to 4, except # where n =2).

| ***S. aureus* ATCC 25923**  6-16 min (melittin)  6-18 min (W2-FL10)  34-50 min (W2-FL10) | **MRSA Xen 30** ^#^  <0.01  <0.1 (ns)  ns | ***S. aureus* RN4220**  <0.001  <0.001  <0.001 |
| --- | --- | --- |
| **MRSA Xen 30**  6-16 min (melittin)  6-18 min (W2-FL10)  34-50 min (W2-FL10) | ***S. aureus* RN4220**  <0.1 (ns)  <0.001  <0.001 |  |

**Figure S1**. Representative examples of the dose response of W2-FL10 towards a variety of Gram-positive bacterial strains. The dose response data (n=3, mean ± standard error of the mean) against each targets cell was fitted with a sigmoidal curve with variable slope to determine the IC_50_ value reported in Table 1.

**Reference list**

1. van Rensburg W, Laubscher WE, Rautenbach M. 2021. High throughput method to determine the surface activity of antimicrobial polymeric materials. MethodsX 8:101593.
